# MPTP-Induced D1R-Dependent Dysregulation of Ventral Hippocampal Newborn Cell Pools and Associated Alterations in the Hypothalamic–Pituitary–Adrenal Axis

**DOI:** 10.3390/brainsci16101098

**Published:** 2026-10-12

**Authors:** Tingting Di, Kai Zhang, Qian Ru, Wenxuan Yin, Yilin Jin, Ling Chen, Hualing Li

**Affiliations:** 1 School of Traditional Chinese Medicine, Faculty of Medicine, Yangzhou University, Yangzhou 225009, China; dididi@yzu.edu.cn (T.D.); 17715821816@163.com (K.Z.); mx320230562@stu.yzu.edu.cn (Q.R.); mx120231236@stu.yzu.edu.cn (W.Y.); 2 Key Laboratory of the Jiangsu Higher Education Institutions for Integrated Traditional Chinese and Western Medicine in Senile Diseases Control, Yangzhou University, Yangzhou 225009, China; 3 The First School of Clinical Medicine, Faculty of Medicine, Yangzhou University, Yangzhou 225009, China; 232210616@stu.yzu.edu.cn; 4 Department of Physiology, Nanjing Medical University, Nanjing 211166, China

**Keywords:** dopaminergic (DAergic) neurons, ventral tegmental area (VTA), ventral hippocampal dentate gyrus (vDG), anteromedial BNST (amBNST), hypothalamic–pituitary–adrenal (HPA) axis

## Abstract

**Objective:** This study investigated how dopaminergic (DAergic) depletion-induced ventral hippocampal neurogenesis triggers hypothalamic–pituitary–adrenal (HPA) axis hyperactivity. **Methods**: Three-month-old male C57BL/6 mice were subjected to systemic 1-Methyl-4-phenyl-1,2,3,6-tetrahydropyridine (MPTP) or ventral tegmental area (VTA) injections of 1-methyl-4-phenylpyridinium ion (MPP+). We observed tyrosine hydroxylase (TH)-positive cells in the midbrain VTA and hippocampal DA content. We examined BrdU-positive cells in the ventral hippocampal dentate gyrus (vDG) on day 7 (D7) and day 28 (D28) after the BrdU injection. The expression of GAD67 and c-Fos-positive cells reflected the activation of GABAergic neurons. We assessed HPA-axis activity based on the levels of CORT, ACTH, and *CRH* mRNA. **Results**: TH immunoreactivity in VTA DAergic neurons and hippocampal DA were markedly reduced on D7 post-MPTP/MPP+ and recovered on D28. The numbers of BrdU-positive cells on D7 and D28 in the vDG were drastically reduced and were remedied by SKF38393 rather than quinpirole. MPTP significantly elevated levels of CORT, ACTH, and *CRH* mRNA on D28. A SKF38393 injection in the vDG, an AMPA injection in the amBNST, or a muscimol injection in the PVN effectively reversed HPA-axis hyperactivity. **Conclusions**: These results indicate that VTA DAergic depletion reduces the number of BrdU-labeled newborn cells within the vDG in a D1R-dependent manner. Given our previous work demonstrating unchanged progenitor cell proliferation but impaired survival of vDG newborn neurons under MPTP insult, we propose that disrupted D1R-dependent maintenance of newborn cell populations impairs vHPC-originated glutamatergic input to the amBNST, with associated alterations in the HPA-axis activity.

## 1. Introduction

The hypothalamic–pituitary–adrenal (HPA) axis plays a pivotal role in the response to diverse stimuli and the integration of multiple physiological processes. Neurons release corticotrophin-releasing hormone (CRH), which stimulates the secretion of adrenocorticotropic hormone (ACTH) from the anterior pituitary and subsequently elevates serum cortisol/corticosterone (CORT) levels. Elevated glucocorticoid levels promote anxiety- and depression-like behaviors, whereas their reduction produces anxiolytic- and antidepressant-like effects [1]. Our previous studies have demonstrated that the hyperactivity of the HPA-axis contributes significantly to the development of affective disorders [2]. Serum CORT levels are markedly increased in patients with Parkinson’s disease (PD) depression [3], implying that the dopaminergic (DAergic) system may modulate the activity of the HPA-axis. 1-methyl-4-phenyl-1,2,3,6-tetrahydropyridine (MPTP)-treated mice (MPTP-mice) exhibit depression-like behaviors accompanied by attenuated negative feedback and the hyperactivity of the HPA-axis, suggesting that this hyperactivity may be involved in the pathogenesis of PD depression.

PD is characterized by the loss of DAergic neurons in the substantia nigra pars compacta (SNpc) and ventral tegmental area (VTA) of the midbrain. DAergic projections from the SNpc to the striatum regulate motor functions, whereas those from the VTA primarily modulate emotional behaviors [4]. Systemic MPTP injures both nuclei; therefore, we used intra-VTA MPP+ microinjection to isolate VTA-specific effects. Although numerous studies have reported that MPTP-induced lesions of midbrain DAergic neurons alter the activity of the HPA-axis, morphological investigations have not identified direct projections from midbrain DAergic neurons to hypothalamic CRH neurons, indicating that midbrain DAergic neurons do not directly regulate the HPA-axis.

The hippocampal volume is significantly reduced in depressed patients, and the therapeutic efficacy of antidepressants is closely linked to the recovery of hippocampal volume [5]. The hippocampus is divided into dorsal and ventral subregions. VTA DAergic neurons mainly project to the ventral hippocampus (vHPC), whose efferents innervate the limbic system to regulate emotions [6], whereas the dorsal hippocampus (dHPC) receives minimal DAergic input. Furthermore, vHPC neurons express DA receptors (DRs) that modulate synaptic plasticity and pathophysiological processes [7,8]. The activation of D1R promotes MPTP-impaired neurogenesis [9]. As a key neuroendocrine region, the vHPC inhibits HPA-axis activity and reduces CORT secretion [10]. Clinical evidence indicates that hippocampal stem cell proliferation is significantly decreased in PD [11]. Impaired adult hippocampal neurogenesis disrupts the homeostasis of the HPA-axis and increases stress sensitivity [12]. VTA DAergic injury may drive HPA-axis hyperactivity and affective dysfunction via disrupted vDG neurogenesis and synaptic transmission.

Notably, hippocampal outputs do not directly innervate CRH neurons in hypothalamic paraventricular nuclei (PVN). Morphological studies have demonstrated that glutamatergic projections from the vHPC target the anteromedial bed nucleus of the stria terminalis (amBNST) [13]. Gama-aminobutyric acid (GABA)-ergic neurons in the BNST receive glutamatergic inputs from the hippocampus and exert critical inhibitory control over the HPA-axis [14]. Accumulating evidence indicates that adult-born dentate granule cells shape downstream limbic circuit output and can modulate neuroendocrine stress responses. Published work has demonstrated that MPTP-mice exhibit depressive-like behaviors, which can be ameliorated via vHPC D1R agonist administration [9]. Altered HPA-axis regulation has also been reported both in MPTP-mice [15] and PD patients [16], implying potential links between DAergic injury, HPA-axis dysregulation and mood disturbances. Although MPTP-evoked VTA DAergic loss disrupts vDG newborn cell populations, whether such newborn cell dysregulation alters downstream amBNST function remains unclarified, as well as its association with HPA-axis dysregulation.

In this study, we test our primary hypothesis: MPTP-triggered VTA DAergic deficit disrupts D1R-dependent maintenance of newborn neuron populations in the vDG. This impairment is proposed to reduce vHPC-derived glutamatergic output to the amBNST, lowering amBNST GABAergic neuronal function and thereby potentially contributing to HPA-axis dysregulation. Accordingly, here we characterize circuit- and time-dependent neuroendocrine changes after MPTP exposure to examine this mechanistic cascade.

## 2. Materials and Methods

### 2.1. Animals

All animal experiments were approved by the Ethical Committee of Yangzhou University (YXYLL-2023-74, 23 February 2023), and all animal handling procedures were fully compliant with the standardized laboratory animal care guidelines issued by the Laboratory Animal Research Institute. Three-month-old male C57BL/6 mice (Yangzhou University Center for Comparative Medicine, Yangzhou, China) were utilized as experimental subjects. Animals were specific-pathogen-free (SPF) grade with good general health status and no pre-existing immune abnormalities. No genetic modification was performed. The animals had not undergone any prior experimental procedures before this study. The mice were housed under consistent environmental conditions (temperature 23 ± 2 °C, humidity 55 ± 5%, and 12:12 h light/dark cycle) in the Animal Research Center of Yangzhou University with free access to food and water. Humane endpoints were predefined, with daily monitoring of animal health, weight and behavior. No animals required early euthanasia during the study. This study was exploratory and not pre-registered. Pre-defined animal exclusion criteria were established before starting experiments: (1) death occurring during surgical/anesthetic procedures; (2) post hoc histological verification demonstrating incorrect stereotaxic cannula/injection placement. Animals meeting either criterion would be excluded from further analysis and replaced with new animals.

### 2.2. Drug Administration

MPTP, MPP+, BrdU, D1R agonist SKF38393, D2R agonist quinpirole, and GABA_A_R agonist muscimol were purchased from MedChemExpress (MCE), and AMPAR agonist AMPA was obtained from Sigma-Aldrich. Drugs were first dissolved in dimethylsulfoxide (DMSO), then further diluted with 0.9% sterile saline or artificial cerebrospinal fluid (aCSF; in mM: 124 NaCl, 1 MgCl_2_, 4.5 KCl, 2 CaCl_2_, 1.2 NaH_2_PO_4_, 10 D-glucose, and 26 NaHCO_3_; pH 7.4) to yield a final DMSO concentration of 0.1% (*v*/*v*).

Systemic administration: The mice were administered daily intraperitoneal (i.p.) MPTP injections (MPTP-mice) at a dose of 25 mg/kg or a vehicle (control mice) for 5 days [9]. A single injection of BrdU (50 mg/kg, i.p.) was delivered to each mouse after the last MPTP injection or single MPP+ injection.

Micro-injection into brain: The mice were anesthetized with ketamine (100 mg/kg, i.p.) and xylazine (10 mg/kg, i.p.) and subsequently fixed onto a Stoelting stereotaxic frame. Following scalp incision, a 2 mm diameter cranial hole was created over the target site with a dental drill. Bilateral implantation of guide cannulas (26-gauge, Plastics One, Roanoke, VA, USA) was then performed. Bilateral drug micro-injection (0.25 μL/side) was performed using a motorized stepping micro-syringe at a constant infusion rate of 0.05 μL/min. All cannula insertions in the present study were performed vertically (orthogonal angle) without angled approach for all brain regions. Stereotaxic coordinates relative to bregma were set as follows: VTA: 3.2 mm posterior, 0.5 mm lateral, 4.69 mm ventral to bregma [17], vDG: 2.3 mm posterior, 1.3 mm lateral, and 2.0 mm ventral to bregma [18]; amBNST: 0.13 mm anterior, 0.8 mm lateral, and 3.5 mm ventral to bregma [19,20]; and PVN: 0.8 mm posterior, 0.2 mm lateral, and 4.0 mm ventral to bregma [21]. Evans blue microinjection was conducted to verify targeting precision for the VTA, vDG, amBNST, and PVN. Post hoc histological verification of cannula trajectories was conducted for all experimental subjects, with injection sites registered against the Franklin–Paxinos mouse brain atlas. No animals were excluded in the present study due to reliable and consistent stereotaxic localization across all subjects. MPP+ (0.36 μg/side) [22], SKF38393 (0.1 μg/side/day), quinpirole (0.1 μg/side/day) [23], AMPA (0.05 μg/side) [24], or muscimol (0.05 μg/side) [25] were used. All stereotaxic microinjections were bilateral; the reported doses for MPP+, SKF38393, quinpirole, AMPA and muscimol refer to the dose per hemisphere. Intra-VTA MPP+ was dosed for selective VTA lesioning while sparing SNpc neurons [15]. SKF38393, quinpirole AMPA and muscimol were used at established in vivo doses to selectively activate D1R, D2R AMPAR and GABA_A_R [15]. Control mice or VTA-control mice received an equal volume of vehicle solution via identical stereotaxic infusion procedures.

### 2.3. Experimental Design and Groups

**Figure 1:**
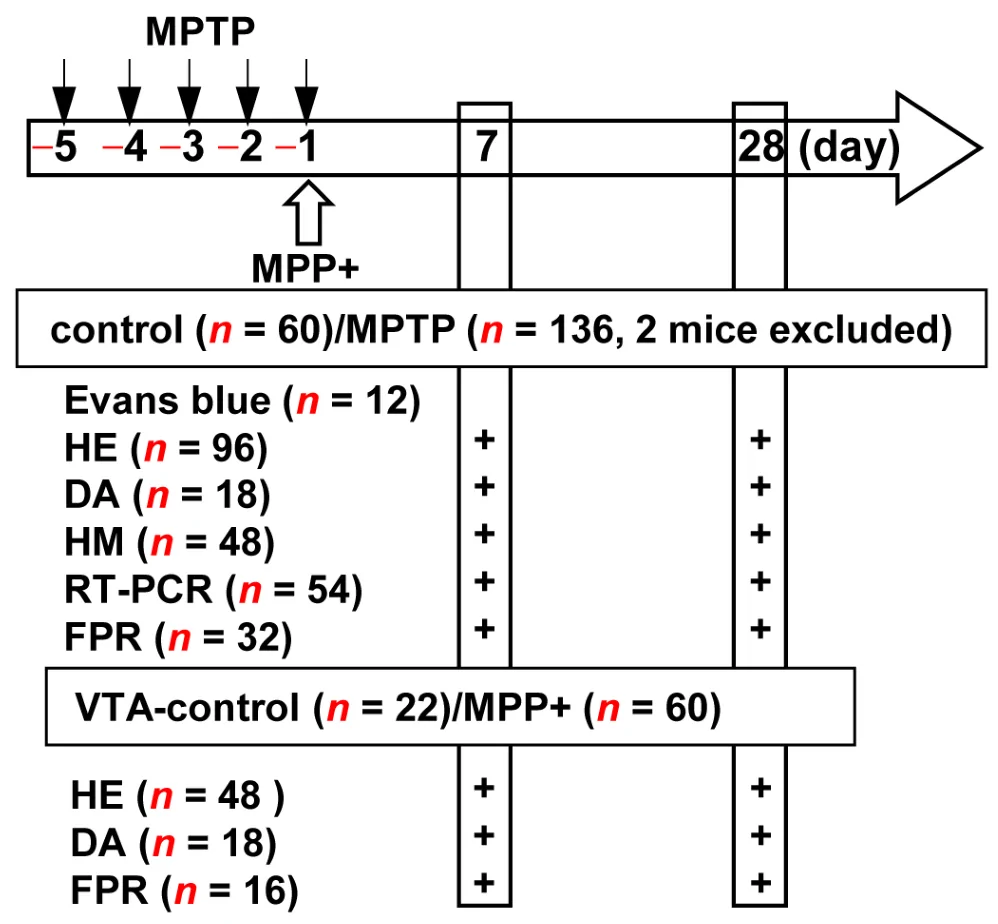
Timecourse of the experimental procedure and sample sizes. HE: histological examination; DA: dopamine content; HM: measurement of hormones; FPR: field potential recording. Note: As some animals provided tissue for multiple assays, sums of subgroup sample sizes exceed the total number of unique animals per group. + indicates the time point for sample collection and detection.

The total number of used mice (*n* = 278) was as follows: control mice (*n* = 60), MPTP-mice (*n* = 146), VTA-control mice (*n* = 22), and MPP+-mice (*n* = 60) (Figure 1). The mice were divided into four experimental groups: The first group was used to verify targeting precision for the VTA, vDG, amBNST, and PVN. The second group was designed to test whether diminished tyrosine hydroxylase (TH) immunoreactivity in VTA DAergic neurons had an effect on BrdU-labeled newborn cell populations within the vDG: TH-positive cells → hippocampal DA content → BrdU-positive cells. The third group was used to examine the influence of reduced TH immunoreactivity in VTA DAergic afferents on vDG synaptic transmission: excitatory postsynaptic potential (fEPSP) and paired-pulse facilitation (PPF). The fourth group (control: *n* = 18, MPTP: *n* = 66) aimed to examine whether VTA DAergic damage was associated with GABAergic inhibition in the amBNST and HPA-axis changes: *GAD67* mRNA, GAD67-positive cells and c-Fos-positive cells → CORT, ACTH and *CRH* mRNA. Two MPTP-mice treated with the vDG injection of SKF38393 had died during the operation and were excluded according to our predefined exclusion criteria, with replacement animals added in a timely manner.

The experimental unit was the individual mouse, uniquely identified via earmark marking. Before any toxin treatment, each mouse was assigned a unique random number using Microsoft Excel 2013; mice were ranked and allocated to corresponding control or experimental groups according to random number order. Researchers performing animal treatments and surgeries were unblinded. Unblinded investigators conducted animal husbandry monitoring, MPTP/intra-VTA MPP+ injections, stereotaxic cannula implantation and tissue harvesting. All quantitative outcome measurements and statistical analyses were completed by an investigator blinded to group allocation.

### 2.4. Histological Examination

Mice were anesthetized with pentobarbital (50 mg/kg i.p.) and subjected to transcardial perfusion with 4% paraformaldehyde (PFA). After perfusion, whole brains were isolated and post-fixed in 4% PFA overnight, followed by cryoprotection in 30% sucrose solution until full tissue submersion. After being fully submerged at the bottom of the 30% sucrose solution, serial brain sections were prepared as follows: the midbrain sections (40 μm) including VTA were obtained from bregma −2.9 mm to −3.9 mm; the vDG sections (40 μm) were obtained from bregma −2.9 mm to −3.9 mm; and amBNST (10 μm) were obtained from bregma 0.62 mm to 0.02 mm. The free-floating sections were incubated at 4 °C overnight with the following primary antibodies: chicken anti-TH (1:1000, Abcam, Cambridge, UK), rat anti-BrdU (1:1000, Millipore, Billerica, MA, USA) mouse anti-GAD67 (1:1000, Millipore), and mouse anti-c-Fos (1:1000, Santa Cruz, Dallas, TX, USA). Following washes, the sections were incubated with the appropriate secondary antibodies for 2 h at 23 ± 2 °C. Cell counting of TH-positive cells in the VTA, BrdU-positive cells in the vDG, as well as GAD67-positive and c-Fos-positive cells in the amBNST was performed using a stereological system, which motorized specimen stage for automated sampling, and the Stereo Investigator software (Microbrightfield, Williston, VT, USA). The number of TH-positive cells (5 sections/mice), GAD67-positive cells (12 sections/mice) and c-Fos-positive cells (12 sections/mice) was averaged to provide an evaluation value per mice [15].

### 2.5. Neurochemical Analyses of DA

Mice were deeply anesthetized with isoflurane and sacrificed via decapitation, followed by rapid whole-brain dissection on ice. Hippocampal tissues were immediately isolated and homogenized via sonication in pre-cooled tissue lysis buffer (0.1 M perchloric acid solution added with 0.4% EDTA) and centrifuged at 12,000× *g* (Beckman Coulter Microfuge, Palo Alto, CA, USA) for 15 min. The DA content in tissue supernatants was analyzed using high-pressure liquid chromatography coupled with electrochemical detection (HPLC-ED). The HPLC setup consisted of a Waters 515 HPLC pump (Waters Corporation, Milford, MA, USA) and an ESA Coulochem 5100 A electrochemical detector. Separation was performed on a Brownlee Spheri-5 RP18 analytical column (4.6 mm × 250 mm, 5 μm particle size; Perkin-Elmer, Waltham, MA, USA) with isocratic elution. The mobile phase comprised 15% (*v*/*v*) methanol and 0.01% sodium octylsulfate, with pH adjusted to 2.65 using phosphoric acid. The mobile phase was filtered through a 0.22 μm membrane and degassed online. The flow rate was set at 1.0 mL/min, and the injection volume was 20 μL. The working-electrode potential of the electrochemical detector was set at +0.40 V versus an Ag/AgCl reference electrode (positive polarity). Under these chromatographic conditions, the retention time for DA was approximately 5.2 min. DA external calibration standards were prepared over the concentration range of 0.5–200 ng/mL to construct standard calibration curves. The limit of detection (signal-to-noise ratio = 3) was 0.12 ng/mL, and the limit of quantitation (signal-to-noise ratio = 10) was 0.40 ng/mL. Intra-assay and inter-assay precision were evaluated using low-, medium-, and high-concentration quality control (QC) samples prepared in blank hippocampal tissue homogenate matrix. The intra-assay coefficient of variation (CV) was less than 6.2%, and the inter-assay CV was less than 8.1%. The spiked recovery of dopamine from hippocampal tissue homogenate ranged from 88.4% to 94.7%. QC procedures: Each analytical batch included solvent blanks, tissue matrix blanks, three levels of matrix-matched QC samples, and serial DA external standards. Samples with concentrations above the upper limit of the calibration range were appropriately diluted in lysis buffer and re-analyzed. DA concentrations in tissue supernatants were calculated by comparing sample peak areas against external DA standards. Total protein concentration of hippocampal tissue lysates was determined using the BCA protein assay kit. Final DA concentrations were normalized with the following formula: normalized DA (ng/g protein) = HPLC-measured DA concentration (ng/mL)/total protein concentration (g/mL).

### 2.6. Field Potential Recording

Brain slices were transferred to a submerged recording chamber continuously perfused with oxygenated artificial cerebrospinal fluid (aCSF), with bath temperature stabilized at 30 ± 1 °C. All pharmacological treatments were delivered via in vivo microinjection to mice from day 3 to day 7 post-MPTP; no drugs were added to the extracellular recording bath during ex vivo slice electrophysiology. A bipolar tungsten stimulating electrode and a glass microelectrode filled with 2 M NaCl (3–5 MΩ) were positioned within the vDG region to stimulate local neuronal fibers and record extracellular field synaptic responses. The signals were recorded using the Axoclamp 2B amplifier, with sampling at 20 kHz and filtering at 10 kHz. The output signals were converted to digital signals using the Digidata 1200 converter (Axon Enterprise, Scottsdale, AZ, USA). Stable baseline required <10% fEPSP-slope variation over 10 consecutive minutes. Recordings were excluded for >10% baseline drift, poor signal-to-noise ratio or deteriorated electrode stability. A stable baseline of fEPSP was recorded for at least 20 min following a stimulus intensity adjusted to elicit ~ 50% of its maximal amplitude. PPF was induced by paired electrical stimuli with inter-pulse intervals (IPIs) set at 25 ms and 50–100 ms.

### 2.7. Reverse Transcription Quantitative Polymerase Chain Reaction (RT-PCR)

Mice were anesthetized with isoflurane and the brains were rapidly removed. The coronal brain slices were cut using a cryostat, and the hypothalamic PVN and amBNST were rapidly harvested using a 15-gauge needle (inner diameter 1.5 mm). Tissues dissected from PVN and amBNST were homogenized for total RNA extraction using TRIzol reagent (Life Technologies, Invitrogen, Camarillo, CA, USA) following the manufacturer’s standard protocols. RNA purity was assessed via A260/A280 ratio. Given the limited tissue mass of PVN and amBNST, a fixed amount of total RNA (100 ng) from each sample was used as the input for reverse-transcription. Purified RNA was reverse-transcribed into complementary DNA (cDNA) using the PrimeScript RT reagent kit (Takara Bio, Kusatsu, Shiga, Japan). Quantitative real-time PCR amplification was performed on an ABI Step One Plus instrument with SYBR Green I fluorescent dye (Takara). All target gene primers were synthesized by Invitrogen, with sequences listed below: *CRH* (forward 5′ -AAGAAATTCAAGGGCTGCGG-3′; reverse 5′-GGAGAAGAGAGCGCCCCTAA-3′), *GAD67* (forward 5′-GCAGATCCTGGTTGACTGTAG-3′; reverse 5′-GAACATATTGGTATTGGCAGTCG-3′) and *GAPDH* (forward 5′-TGCCCCCATGTTTGTGATG-3′, reverse 5′-TGTGGTCATGAGCCCTTCC-3′). The amplicon lengths and experimentally determined primer amplification efficiencies were as follows: *CRH* (124 bp, 97.8%), *GAD67* (138 bp, 98.5%), and *GAPDH* (110 bp, 99.1%). Amplification efficiencies were calculated from serial cDNA dilution standard curves, and all values fell within the acceptable range of 90–110%. *GAPDH* Ct values were compared across control, MPTP, VTA-control and MPP+ groups. No significant inter-group difference in *GAPDH* Ct was detected (F_(3,20)_ = 0.1080, *p* = 0.9545, *n* = 6, Supplementary Figure S1), confirming stable *GAPDH* mRNA expression under the present experimental conditions. Relative gene expression levels were normalized to the housekeeping gene GAPDH and calculated using the 2^−ΔΔCt^ comparative threshold cycle method. Each biological sample was analyzed in technical triplicates to generate average Ct values for subsequent quantification.

### 2.8. Hormone Measurement

Basal level of CORT exhibits a sinusoidal pattern during one day and the highest level appears just prior to the onset of waking in mice (18:00–20:00) [25]. To measure basal CORT and ACTH levels, approximately 300 μL of orbital venous blood was collected from mice (18:00 to 19:00) [26]. Although orbital blood collection may trigger mild acute stress if prolonged, the strict short sampling time (within 90 s) used in this study limited this confounding effect. Serum was obtained via centrifugation at 4 °C (Angle Rotor; Thermo Scientific, Waltham, MA, USA) and stored at −20 °C until enzyme-linked immunosorbent assay (ELISA) was performed. Serum CORT concentrations were determined using an ELISA kit (Cat# RE52211, IBL International GmbH, Hamburg, Germany). The assay dynamic range for CORT is 1.68–240 nmol/L, analytical sensitivity is 0.589 nmol/L, intra-assay CV ranges from 3.3 to 7.7%, inter-assay CV ranges from 5.0 to 10.8%. ACTH levels were quantified via an ELISA kit (Cat# BP00912, Baipeng, Shanghai, China). The detection range for ACTH is 3.12–200 pg/mL, with a minimum detectable dose of 1.71 pg/mL; intra-assay CV < 10%, inter-assay CV < 10%. Serum samples were undiluted for both CORT and ACTH assays. For samples with signals exceeding the upper calibrator limit, serum was diluted and re-measured and final CORT concentrations were corrected by the dilution factor.

### 2.9. Data Analysis/Statistics

Grubbs’ test was used to screen for outliers within each experimental group before performing group comparisons, with a significance threshold set at α = 0.05. No outliers were detected across all datasets, and therefore no raw data points were excluded from subsequent statistical analyses. All raw experimental data were sorted and visualized using GraphPad Prism 8.0 software. All quantitative results were presented as the mean ± standard deviation (SD). Statistical calculations were completed using SPSS 25.0 software (SPSS Inc., Chicago, IL, USA). Normality was assessed by Shapiro–Wilk test and homogeneity of variance by Levene’s test. Parametric tests were applied when assumptions were satisfied; nonparametric tests would be adopted if assumptions failed. All datasets in this study met parametric test assumptions. One-way or two-way analysis of variance (ANOVA) was utilized to evaluate intergroup differences, followed by Bonferroni or Šidák post hoc tests for pairwise multiple comparisons where appropriate. Effect sizes and 95% confidence intervals (95% CIs) were calculated for all primary mechanistic comparisons. Statistical significance was defined as *p* < 0.05 for all analyses.

## 3. Results

### 3.1. Effects of MPTP/MPP+ on TH-Positive Cells in the VTA and DA Content in the Hippocampus

**Figure 2:**
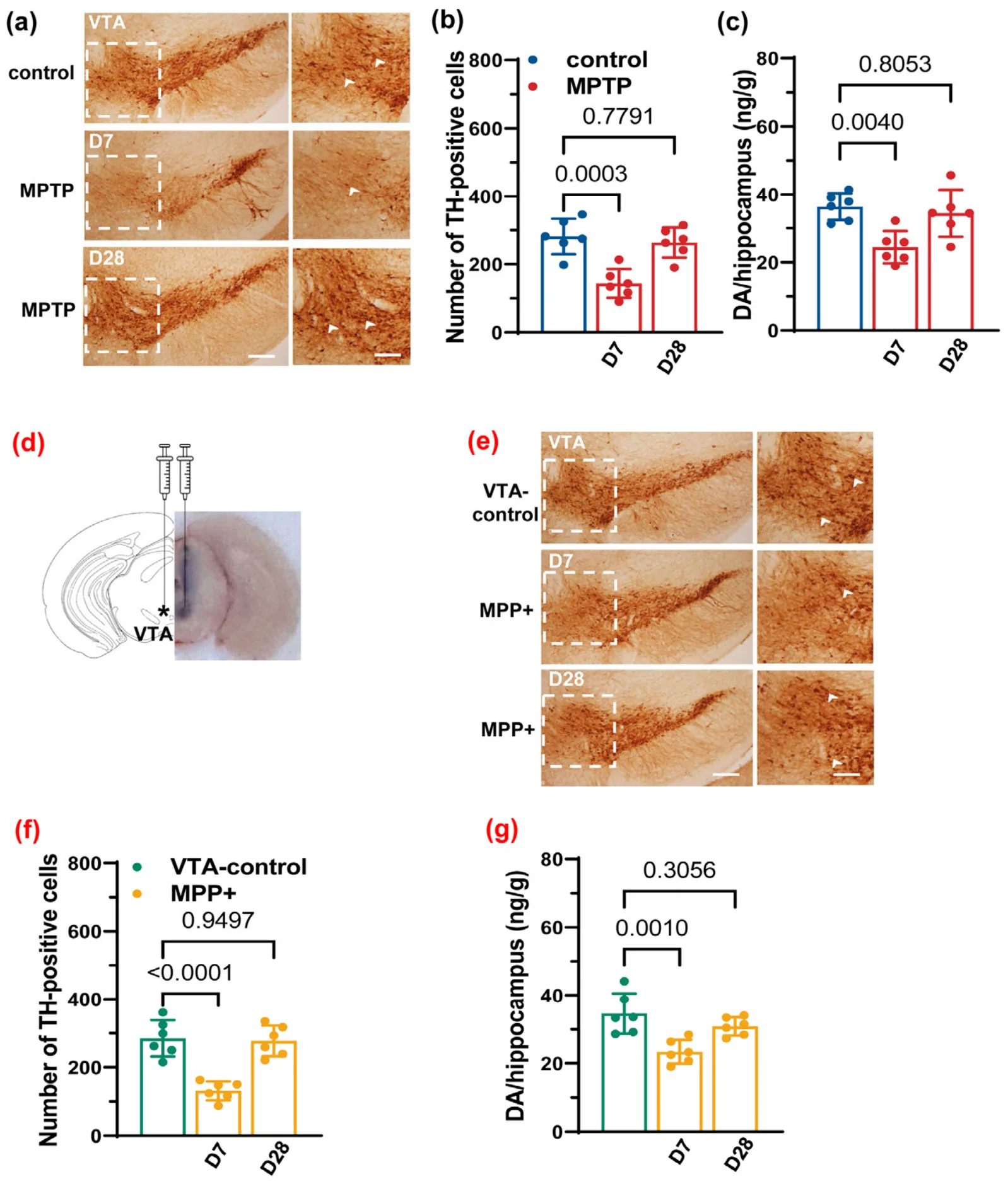
Effects of MPTP/MPP+ on TH-positive cells in the VTA and DA content in the hippocampus. (**a**,**b**) Representative photomicrographs of TH immunohistochemical staining and number of TH-positive cells in the VTA on D7 or D28 after the last injection of MPTP (one-way ANOVA). Arrows indicate TH-positive cells. Scale bar: 40 μm (**left**), 100 μm (**right**). (**c**) DA content in the hippocampus on D7 or D28 after the last injection of MPTP (one-way ANOVA). (**d**) Schematic diagram of VTA (**left** panel) and image of the VTA injected with Evans blue (**right** panel). Star indicates the site of injection. (**e**,**f**) Representative photomicrographs of TH immunohistochemical staining and number of TH-positive cells in the VTA on D7 or D28 after or a single VTA injection of MPP+ (one-way ANOVA). Arrows indicate TH-positive cells. Scale bar: 40 μm (**left**), 100 μm (**right**). (**g**) DA content in the hippocampus on D7 or D28 after a single VTA injection of MPP+ (one-way ANOVA).

Measurements were targeted to the VTA instead of the SNpc, as VTA DAergic circuits are the primary mediators of PD-relevant limbic–neuroendocrine dysfunction. TH immunoreactivity in the VTA and the hippocampal DA content were examined on day 7 (D7) and day 28 (D28) after the last MPTP injection or single MPP+ injection. The number of TH-positive cells was reduced in the MPTP-mice compared to control mice on D7 (*p* = 0.0003, *n* = 6 mice, Figure 2a,b). In addition, the DA content in the hippocampus (*p* = 0.0040, *n* = 6 mice, Figure 2c) was significantly lower than in the control mice. By contrast, the number of TH-positive cells (*p* = 0.7791, *n* = 6 mice) and hippocampal DA content (*p* = 0.8053, *n* = 6 mice) returned to normal levels after 28 days post-MPTP.

Intra-VTA MPP+ microinjection, or vehicle-treated VTA-control mice (Figure 2d, *n* = 6 mice), was used to selectively lesion VTA neurons, disentangle VTA-specific effects from confounds of concomitant SNpc injury in MPTP-mice, and probe functional VTA–hippocampal connections. Compared with the VTA-control mice, the number of TH-positive cells (*p* < 0.0001, *n* = 6 mice, Figure 2e,f) and the hippocampal DA content (*p* = 0.0010, *n* = 6 mice, Figure 2g) were decreased in MPP+-mice on D7. Similarly, there were no significant differences in the number of TH-positive cells (*p* = 0.9497, *n* = 6 mice) and hippocampal DA content (*p* = 0.3056, *n* = 6 mice) between MPP+-mice and VTA-control mice on D28. These results suggest that 7 days after MPTP/MPP+ administration, the number of TH-positive cells in the VTA and hippocampal DA content were significantly reduced, but these changes will recover after 28 days.

### 3.2. Reduced VTA TH-Immunoreactive Afferent Signaling Impairs D1R-Mediated Populations of BrdU-Labeled Newborn Cells in the vDG

**Figure 3:**
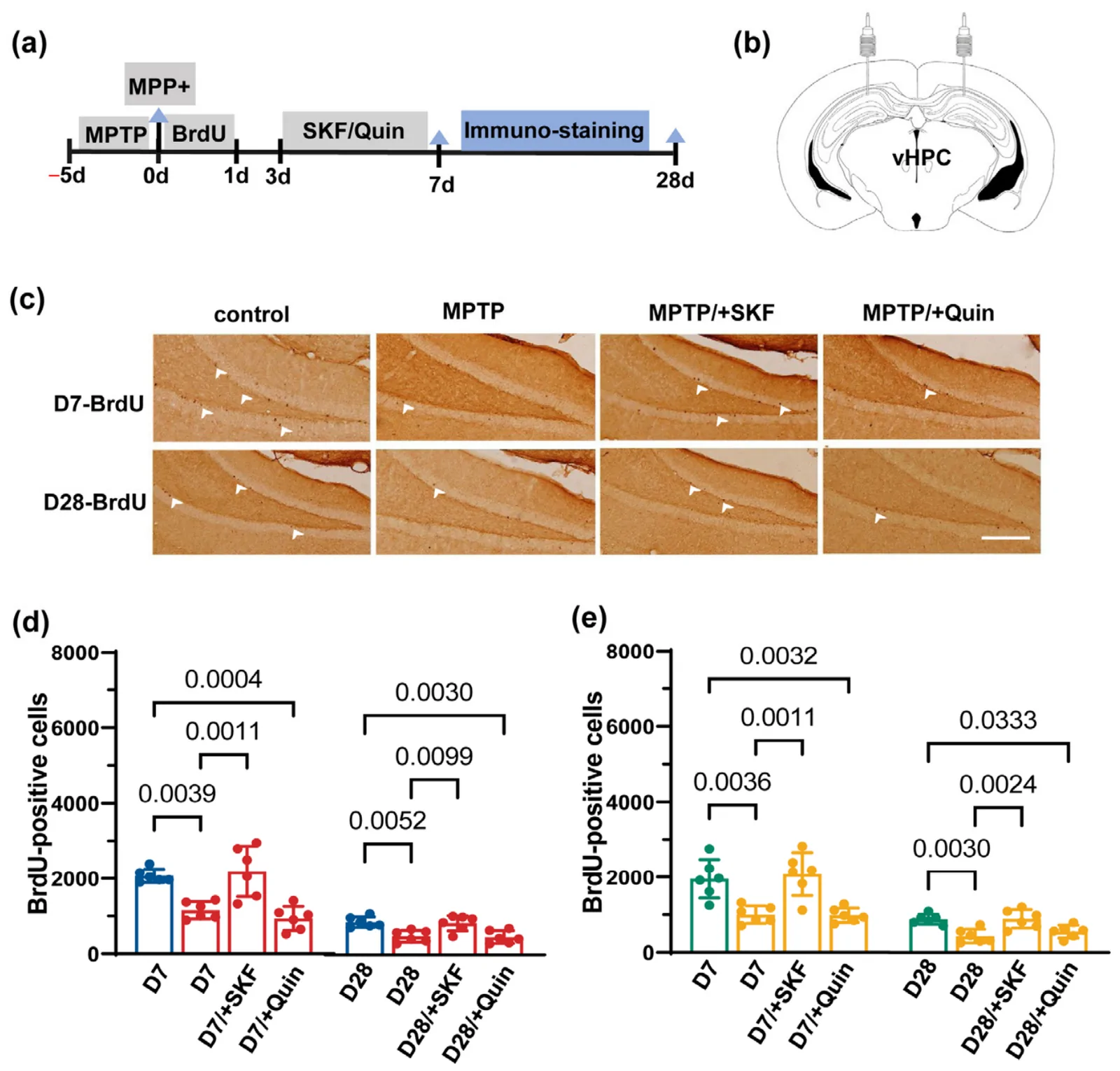
Reduced VTA TH-immunoreactive afferent signaling impairs D1R-mediated populations of BrdU-labeled newborn cells in the vDG. (**a**) The time course of the experimental procedure. (**b**) Schematic representation of bilateral local microinjection into the vHPC. (**c**) Representative photomicrographs of D7/D28-BrdU-positive cells (white arrows) in vDG. Scale bar: 50 μm. (**d**,**e**) Bar graph shows the mean number of D7/D28-BrdU-positive in vDG. MPTP-mice/MPP+-mice were treated with intra-vHPC injection of D1R agonist SKF38393 (SKF) or D1R agonist quinpirole (Quin) (one-way ANOVA).

The BrdU was injected on the day after the last MPTP injection or single MPP+ injection to label progenitor cells that proliferate after MPTP-induced DAergic injury (Figure 3a). BrdU-positive cells were examined on D7 and D28 post-BrdU injection to assess the persistence of this injury-induced newborn cell populations. Consistent with previous research results [9], the numbers of BrdU-positive cells on D7 and D28 in the vDG of the MPTP-mice were significantly decreased (D7: *p* = 0.0039, D28: *p* = 0.0052, *n* = 6 mice, Figure 3c,d). From day 3 to 7 after the last MPTP injection, the vHPC was administered with the D1R agonist SKF38393 and D2R agonist quinpirole, respectively (Figure 3b). The injection of the D1R agonist may have restored the numbers of BrdU-positive cells on D7 and D28 (D7: *p* = 0.0011, D28: *p* = 0.0099, *n* = 6 mice), but the D2R agonist had no effect (D7: *p* = 0.7794, D28: *p* = 0.9951, *n* = 6 mice). Likewise, we observed that the number of BrdU-positive cells in the vDG was significantly decreased in MPP+-mice on D7 and D28 (D7: *p* = 0.0036, D28: *p* = 0.0030, *n* = 6 mice, Figure 3e). This reduction was reversed by the D1R agonist (D7: *p* = 0.0011, D28: *p* = 0.0024, *n* = 6 mice). These data indicate that diminished signaling from TH-immunoreactive VTA afferents is associated with reduced BrdU-labeled newborn cell numbers in the vDG, and that this phenotype can be rescued by D1R activation. As previously reported [9], MPTP impairs newborn neuron survival without affecting hippocampal progenitor proliferation; our findings thus suggest a D1R-dependent maintenance of these newborn cell populations.

### 3.3. Impaired Excitatory Synaptic Efficacy Accompanies Altered Newborn Cell Populations in the vDG

**Figure 4:**
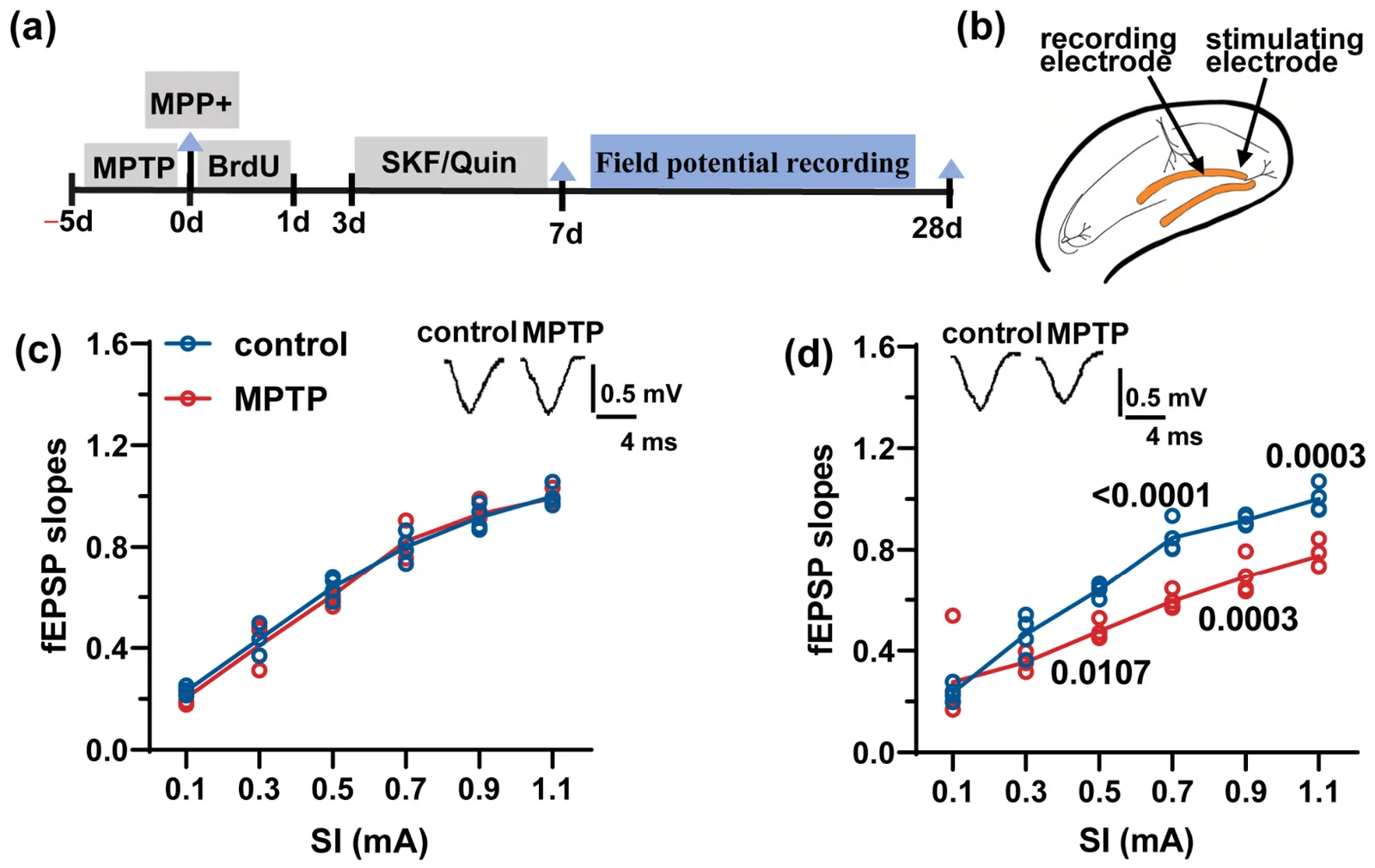

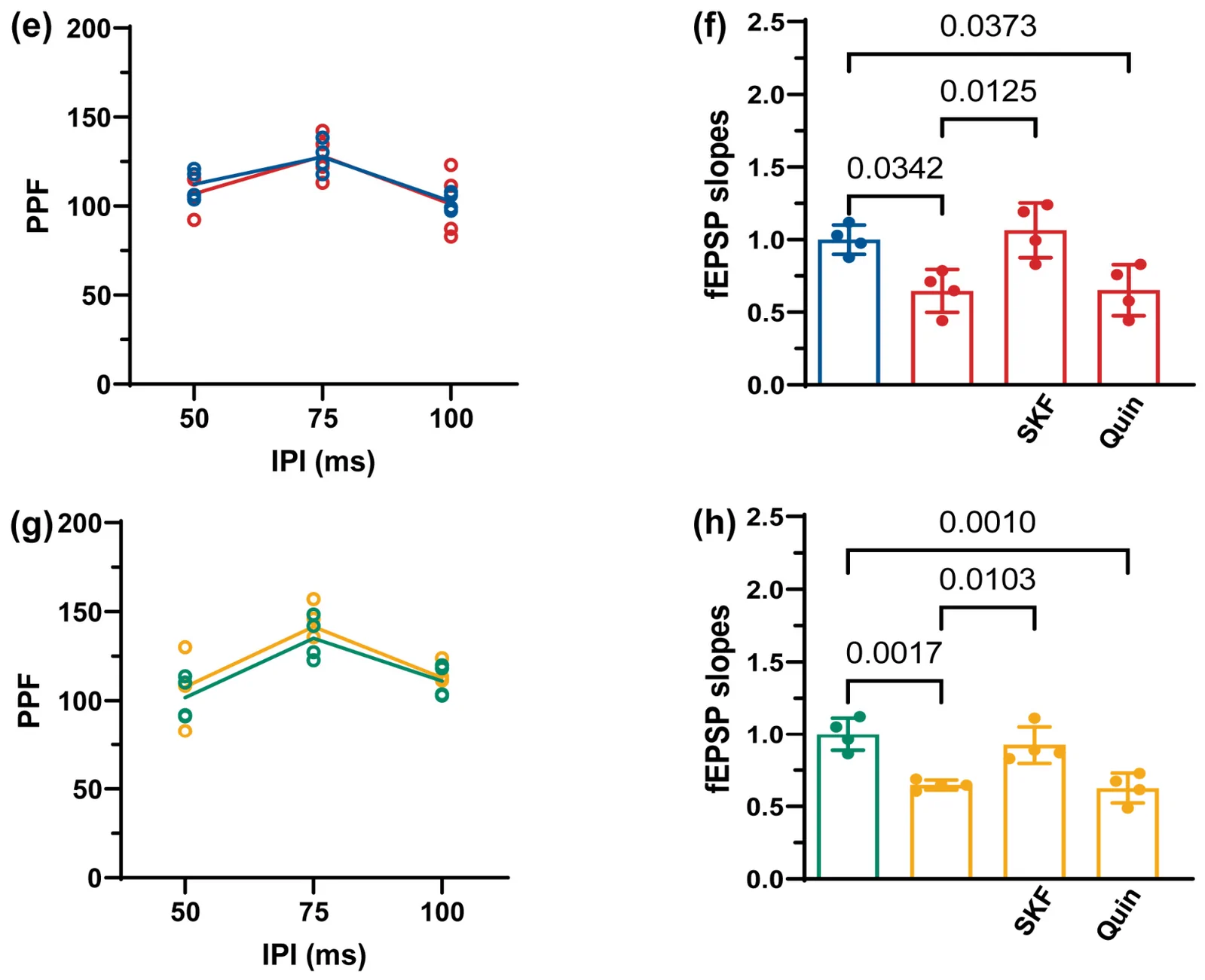
Impaired excitatory synaptic efficacy accompanies altered newborn cell populations in the vDG. (**a**) The time course of the experimental procedure. (**b**) Schematic illustrating sites of stimulating glutamatergic afferents and recording fEPSPs in the vDG. (**c**,**d**) Input/output (I-O) curve on D7 or D28 after post-MPTP. Each point represents the group mean value of fEPSP slopes against the stimulus intensity from 0.1 mA to 1.1 mA (two-way ANOVA). (**e**,**g**) PPF of fEPSP. The paired-pulse ratio (PPR, %) of the fEPSP slopes was plotted against IPIs ranging from 50 ms to 100 ms (two-way ANOVA). (**f**,**h**) Bar graphs of the fEPSP slopes in control slices, MPTP slices, VTA-control slices and MPP+ slices treated with intra-vHPC injection of SKF38393 (SKF), quinpirole (Quin) (one-way ANOVA).

Newborn cells in the vDG directly affect the activity of the CA3–CA1 neural circuit, thus we examined excitatory synaptic transmission in the vDG using field potential recording with local stimulation applied to the vDG (Figure 4a,b). On D7 after the last MPTP injection, there was no significant change in fEPSP slopes (*p* = 0.5157, *n* = 4 mice, Figure 3c). However, the fEPSP slopes against stimulating intensities (SI, 0.1–1.1 mA) in the slices obtained from MPTP-mice (MPTP-slices) were reduced compared with those in the control slices on D28 (0.5 mA: *p* = 0.0107, 0.7 mA: *p* < 0.0001, 0.9 mA: *p* = 0.0003, 1.1 mA: *p* = 0.0003, *n* = 4 mice, Figure 4d). To evaluate the presynaptic capability for glutamate release, paired-pulse facilitation (PPF) of fEPSP was induced using paired-pulse stimulation (0.5 mA) at inter-pulse intervals (IPIs) of 50–100 ms. The result showed that there was no significant change in PPF values (*p* = 0.6608, *n* = 4 mice, Figure 4e). The reduced fEPSP slopes in the vDG of the MPTP-slices could be recovered by the vHPC injection of D1R agonist SKF38393 (*p* = 0.0125, *n* = 4 mice, Figure 4f), but the D2R agonist had no effect (*p* > 0.9999).

In addition, there was no difference in PPF values on D28 after the MPP+ injection (*p* = 0.3777, *n* = 4 mice, Figure 4g), and the fEPSP slopes in the vDG were also significantly smaller than those of the control slices (*p* = 0.0017, *n* = 4 mice, Figure 4h). The vHPC injection of D1R agonist SKF38393 could restore the fEPSP slopes on D28 after the MPP+ injection (*p* = 0.0103, *n* = 4 mice), but the D2R agonist had no effect (*p* = 0.9891, *n* = 4 mice). The unchanged PPF indicated that presynaptic glutamate release probability was not altered in MPTP/MPP+ mice. Together with the reduced fEPSP slope, these results demonstrate impaired excitatory synaptic transmission in vDG that arises independently of changes in presynaptic release probability. Collectively, these findings suggest that disrupted VTA DAergic signaling to the vHPC is associated with reduced D1R-dependent maintenance of vDG newborn cell populations, accompanied by altered vDG synaptic transmission.

### 3.4. Impaired vDG Excitatory Synaptic Transmission Alters GAD67 Expression and Neuronal c-Fos Activation in the amBNST

**Figure 5:**
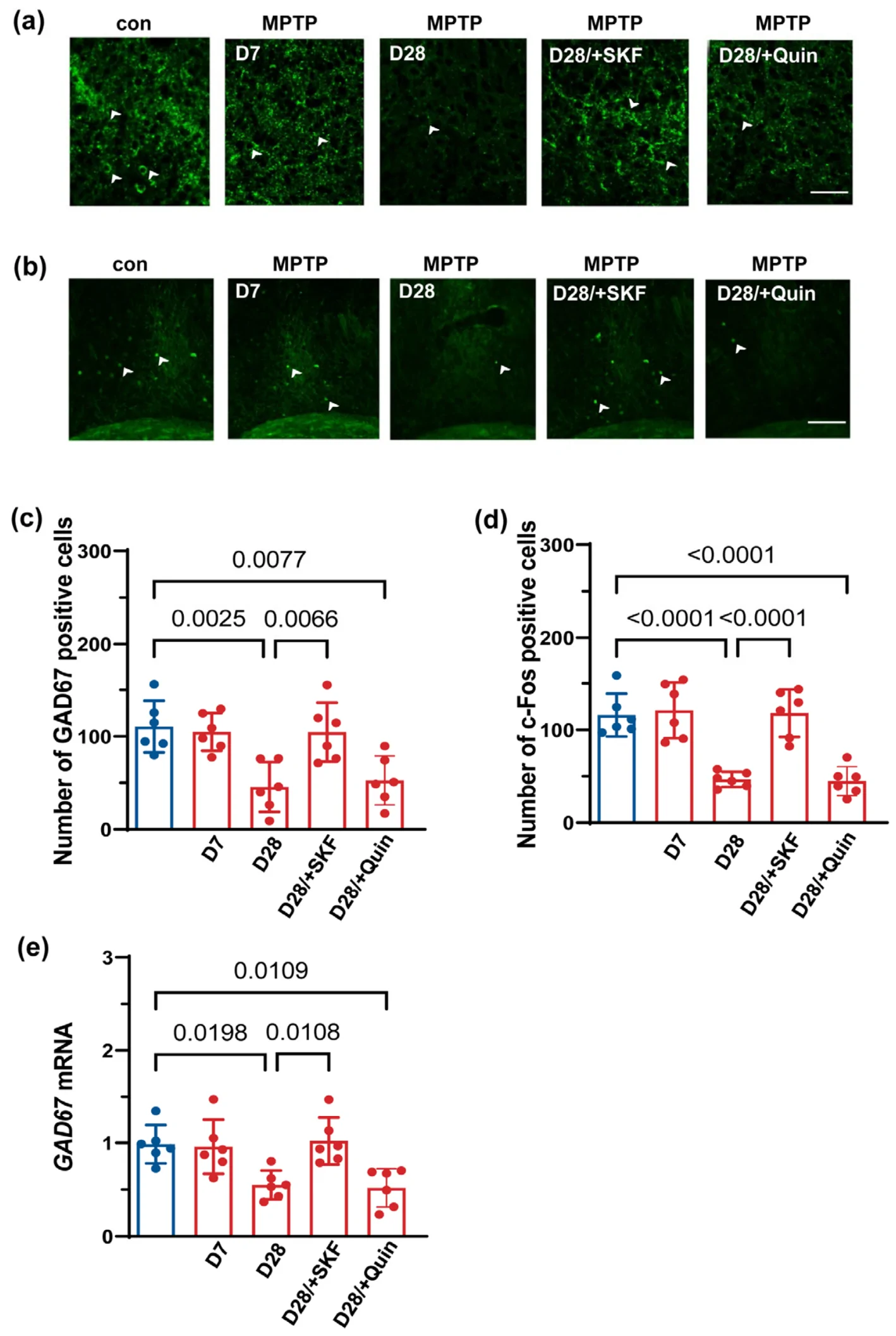
Impaired vDG excitatory synaptic transmission alters GAD67 expression and neuronal c-Fos activation in the amBNST. (**a**,**c**) Representative photomicrographs of GAD67 immunohistochemical staining and the number of GAD67-positive cells in the amBNST on D7 or D28 after the last injection of MPTP (one-way ANOVA). MPTP-mice were treated with intra-vHPC injection of SKF38393 (SKF), quinpirole (Quin). Scale bar: 40 μm. Arrows indicate GAD67-positive cells. (**b**,**d**) Representative photomicrographs of c-Fos immunohistochemical staining and the number of c-Fos-positive cells in the amBNST (one-way ANOVA). Scale bar: 40 μm. Arrows indicate c-Fos-positive cells. (**e**) Levels of *GAD67* mRNA in the amBNST (one-way ANOVA).

DAergic innervation of the BNST is regionally heterogeneous; prominent DAergic projections target the lateral BNST, whereas available anatomical work has not specifically characterized DAergic afferents to the amBNST [27,28]. The amBNST receives glutamatergic nerve fibers from the vHPC [13]. Further experiments examined the impact of impaired synaptic transmission in the vDG on GAD67 expression and neuronal activation in the amBNST. The results revealed that 28 days after the MPTP injection, the amount of GAD67-positive (*p* = 0.0025, *n* = 6 mice, Figure 5a,c) and c-Fos-positive cells (*p* < 0.0001, *n* = 6 mice, Figure 5b,d) was reduced. The level of *GAD67* mRNA was also lower in the amBNST (*p* = 0.0198, *n* = 6 mice, Figure 5e). However, there were no significant changes in *GAD67* mRNA, GAD67-positive and c-Fos-positive cells on D7 (GAD67-positive cells: *p* = 0.9957, c-Fos-positive cells: *p* = 0.9940, *GAD67* mRNA: *p* = 0.9994, *n* = 6 mice). The administration of the D1R agonist into the vHPC could restore the level of *GAD67* mRNA, as well as GAD67-positive and c-Fos-positive cells in the amBNST (GAD67-positive cells: *p* = 0.0066, c-Fos-positive cells: *p* < 0.0001, *GAD67* mRNA: *p* = 0.0108, *n* = 6 mice), but the D2R agonist had no significant effect (GAD67-positive cells: *p* = 0.9908, c-Fos-positive cells: *p* = 0.9999, *GAD67* mRNA: *p* = 0.9990, *n* = 6 mice). These results suggest that MPTP-induced GAD67 expression and overall neuronal activation in the amBNST were partially reversed by intra-vDG D1R agonist treatment.

### 3.5. Impaired D1R-Dependent Maintenance of vDG Newborn Cell Populations Is Associated with Elevated HPA-Axis Activity

**Figure 6:**
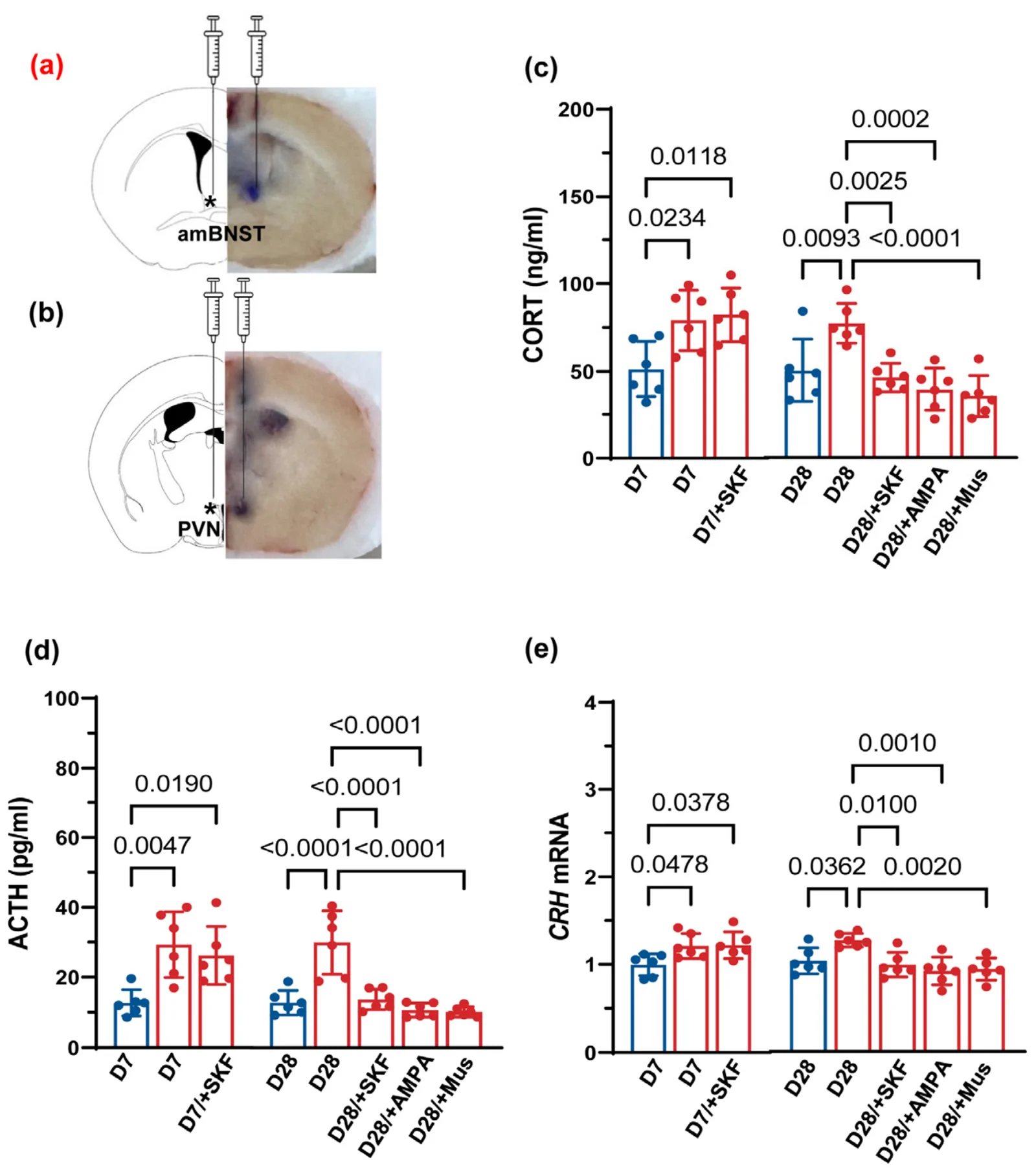
Impaired D1R-dependent maintenance of vDG newborn cell populations is associated with elevated HPA-axis activity. (**a**,**b**) Schematic diagram of amBNST/PVN (**left** panel) and image of the amBNST/PVN injected with Evans blue (**right** panel). Star indicates the site of injection. (**c**,**d**) Levels of serum CORT and ACTH in control mice and MPTP-mice on D7 and D28 after the last injection of MPTP. MPTP-mice were treated with intra-vHPC injection of SKF38393 (SKF) or intra-amBNST injection AMPA or intra-PVN injection of muscimol (Mus) (one-way ANOVA and two-way ANOVA). (**e**) Level of *CRH* mRNA in the PVN on D7 and D28 after the last injection of MPTP (one-way ANOVA and two-way ANOVA).

To confirm whether the impaired D1R-dependent maintenance of vDG newborn cell populations is associated with changes in physiological indices of HPA-axis activity, we assessed the levels of CORT, ACTH, and *CRH* mRNA in the PVN on D7 and D28 after the last MPTP injection. The results showed that the levels of CORT (*p* = 0.0234, *n* = 6 mice, Figure 6c) and ACTH (*p* = 0.0047, *n* = 6 mice, Figure 6d), as well as *CRH* mRNA (*p* = 0.0478, *n* = 6 mice, Figure 6e) in the PVN were significantly higher in MPTP-mice than control mice on D7. However, the intra-vHPC administration of the D1R agonist failed to alter the levels of CORT, ACTH, and *CRH* mRNA in the PVN (CORT: *p* = 0.9360, ACTH: *p* = 0.7659, *CRH* mRNA: *p* = 0.9917, *n* = 6 mice), which implied that D1R-associated neurogenesis is unlikely to drive early HPA-axis dysregulation at this time point. The levels of CORT, ACTH and *CRH* mRNA in the PVN were significantly enhanced in MPTP-mice on D28 (CORT: *p* = 0.0093, ACTH: *p* < 0.0001, *CRH* mRNA: *p* = 0.0362, *n* = 6 mice). Consistent with this temporal dissociation, two-way ANOVA (time × treatment) was further performed to formally compare the magnitude of HPA-axis activation across the two time points. No significant time × treatment interaction was observed for CORT (F_(1,20)_ = 0.0055, *p* = 0.9412), ACTH (F_(1,20)_ = 0.0112, *p* = 0.9133), or *CRH* mRNA (F_(1,20)_ = 0.0522, *p* = 0.8214).

To further examine whether vDG newborn cell populations and amBNST circuit changes are associated with HPA-axis hyperactivity in MPTP-treated mice, we administered intra-vHPC of the D1R agonist SKF38393 from day 3 to 7 after the last MPTP injection, a single micro-injection of the glutamate AMPAR agonist AMPA into the amBNST (Figure 6a) or the GABA_A_R agonist muscimol into the PVN (Figure 6b) on D28. The results revealed that the D1R agonist could restore the levels of CORT and ACTH, as well as *CRH* mRNA in the PVN (CORT: *p* = 0.0025, ACTH: *p* < 0.0001, *CRH* mRNA: *p* = 0.0100, *n* = 6 mice). Similarly, the injection of the AMPAR agonist AMPA or the GABA_A_R agonist muscimol could restore the levels of CORT and ACTH as well as *CRH* mRNA (AMPA: CORT: *p* = 0.0002, ACTH: *p* < 0.0001, *CRH* mRNA: *p* = 0.0010; Mus: CORT: *p* < 0.0001, ACTH: *p* < 0.0001, *CRH* mRNA: *p* = 0.0020, *n* = 6 mice). These results suggest that GABA_A_R-dependent inhibitory control over the HPA-axis can be restored pharmacologically at the PVN, consistent with a model in which amBNST-originated GABAergic signaling contributes to HPA-axis regulation.

## 4. Discussion

The present study reveals that MPTP disrupts vDG newborn neurons, and D1R activation can ameliorate this deficit. Our observations raise a hypothetical mechanistic cascade connecting VTA-DA insufficiency, altered vDG neurogenesis, changed amBNST GAD67 and c-Fos immunoreactivity, and dysregulated HPA-axis. To our knowledge, this work provides initial correlative evidence for this putative circuit; however, a complete functional circuit remains to be formally validated in future investigations. This conclusion is based on the following observations: (1) The numbers of D7 and D28 BrdU-positive cells in the vDG of MPTP-mice and MPP+-mice were significantly lower than that of the control mice. The activation of D1R could ameliorate the reduction in BrdU-positive cells. (2) The glutamatergic synaptic output in the vDG was impaired on D28 after the last MPTP/MPP+ injection, which was also mitigated by the activation of D1R. (3) On D28 after the last MPTP injection, the number of GAD67-positive cells and c-Fos-positive cells in the amBNST was significantly reduced, and the level of *GAD67* mRNA was also decreased but could be partially reversed by the activation of D1R. (4) The vHPC injection of the D1R agonist, the amBNST injection of the AMPAR agonist, or the PVN injection of the GABA_A_R agonist in MPTP-mice was able to mitigate the enhanced HPA-axis activity.

Systemic MPTP and intra-VTA MPP+ are complementary but not equivalent models. Systemic MPTP reproduces PD-like midbrain DAergic injury for studying limbic–neuroendocrine phenotypes under disease-relevant conditions, though it damages both the SNpc and VTA. By contrast, intra-VTA MPP+ is a mechanistic tool that selectively injures only the VTA, to test whether isolated VTA damage alone can trigger downstream hippocampal changes. Results from one model cannot be directly generalized to the other. Our functional results from both systemic MPTP and intra-VTA MPP+ lesion demonstrate that VTA DAergic neuron damage is sufficient to reduce the persistence of BrdU-labeled newborn cell populations in the vDG. While we observe reduced total hippocampal DA after VTA injury, our data cannot distinguish monosynaptic VTA–vDG innervation versus indirect polysynaptic pathways. Our interpretation is supported by published anatomical evidence reporting DA nerve fibers from the VTA project to the newly generated nerve cells in the vDG [29], and whereas the dorsal hippocampal dentate gyrus (dDG) receives minimal DAergic inputs [30]. Numerous newly generated granule neurons in the vDG express D1R and D2R [10]. Clinical data demonstrate that the stem cell proliferation capacity in the hippocampus of PD patients is significantly diminished [11]. Consistent with the prior work by Zhang et al. [9], we observed that TH-positive cells in VTA DAergic neurons and the DA content in the hippocampus were significantly reduced on D7 after 5 consecutive days of intraperitoneal injections of MPTP or VTA injections of MPP+. Notably, DA levels spontaneously recovered to the control baseline after 28 days post-MPTP, reflecting earlier observations from Petroske et al. [31]. We extended these findings using a single-dose MPP+ injury model to further confirm early DA loss on D7 followed by DA restoration on D28, verifying that subacute DAergic toxin exposure triggers only temporary VTA DAergic depletion rather than permanent deficiency. Notably, rebound TH expression does not indicate full functional recovery of VTA DAergic neurons. In this subacute MPTP model, TH levels may recover while long-range circuit signaling remains impaired, maintaining downstream neuroendocrine and circuit abnormalities. Zhang et al. [9] found that the number of D7, D14, and D21 BrdU-positive cells in the vDG of MPTP-mice was significantly reduced. As MPTP does not alter progenitor cell proliferation, reduced TH immunoreactivity among VTA DAergic neurons appears to compromise the survival of newborn neurons after their generation rather than progenitor proliferation. Our data further validated consistent losses of D7 and D28 newborn neurons in both MPTP and MPP+ models. The injection of the D1R agonist from 3 to 7 days after the last MPTP injection or single MPP+ injection could improve the reduction in D7 and D28 BrdU-positive cells, but the D2R agonist had no effect.

Existing studies have demonstrated that newly generated neurons in the DG can improve the excitatory neural synaptic transmission between the olfactory cortex and dentate gyrus-CA3 circuit [32]. Reversible optogenetic silencing of adult-born dentate granule cells perturbs hippocampal circuit function [33]. Conversely, chronic optogenetic stimulation of the vDG expanded the newborn neuron pool and elevated excitatory postsynaptic currents (EPSCs) in CA3. Our study determined that there was no significant change in the excitatory neural synaptic transmission between the medial perforant pathway of the olfactory cortex and the granule cells of the vDG on D7 post-MPTP/MPP+. However, on D28 after the last MPTP or MPP+ injection, this excitatory neural synaptic transmission was significantly lower than that of the control group, but there was no significant change in the glutamate release from the medial perforant fibers of the entorhinal cortex. The administration of the D1R agonist from 3 to 7 days after the last MPTP injection or MPP+ injection could restore the fEPSP slopes in the vDG, suggesting that the D1R-mediated survival of newly generated neurons in the vDG has a positive effect on the excitability of granule cells in this area. Despite normalized bulk hippocampal DA concentrations at D28, D1R-dependent maintenance of vDG BrdU-labeled newborn cell populations and downstream circuit function remain persistently impaired. This important dissociation represents a key novel observation of the present work. One plausible interpretation is that the early transient DAergic perturbation occurring at D7 initiates long-term disturbance of vDG newborn cell homeostasis. Recovery of adult-born granule-cell pools requires prolonged periods for cell survival, maturation and circuit integration. Even when total hippocampal DA content returns to baseline by D28, D1R-dependent circuit outputs cannot be rapidly restored. The mechanism linking mature newborn neurons to intact synaptic transmission remains incompletely defined in the present dataset, which warrants further mechanistic interrogation. A prevailing candidate mediator is the brain-derived neurotrophic factor (BDNF), abundantly secreted by adult-born hippocampal neurons [34]. BDNF facilitates dendritic spine maturation, amplifies excitatory synaptic transmission, and relieves GABAergic local circuit inhibition to elevate overall neuronal excitability [35,36,37]. Consistently, diabetic mouse models with impaired hippocampal BDNF release display suppressed hippocampal circuit activity [38]. Bekinschtein et al. [37] proposed that fully integrated mature newborn neurons exert circuit-modulatory effects in synergy with BDNF signaling. Therefore, we hypothesize that the depletion of newborn neurons can reduce local BDNF bioavailability within the vDG, weakening granule cell output along the vDG–CA3–CA1 excitatory circuit and disrupting downstream extra-hippocampal stress regulatory pathways. Loss- and gain-of-function experiments targeting BDNF secretion specifically in newborn neurons should be performed in the future to directly test this hypothesis.

The vHPC is a key brain region of the neuroendocrine system, capable of suppressing HPA-axis activity and reducing CORT release [10]. Damage to adult hippocampal neurogenesis disrupts HPA-axis regulation and enhances the stress sensitivity of the axis [39]. Chronic stress inhibits hippocampal neurogenesis, increases serum CORT levels, and produces depression-like behaviors [40,41]. Anatomical mapping confirms selective monosynaptic glutamatergic projections from the vHPC exclusively to amBNST GABAergic neurons, and the dorsolateral BNST (dlBNST) essentially receives no nerve inputs from the hippocampus [42,43]. AmBNST nerve fibers project to neurons in the PVN, and these neurons are mainly GABA inhibitory [44,45]. High-frequency stimulation of the vHPC significantly increases the excitability of amBNST neurons [46]. In the literature, it has been reported that by blocking the excitatory nerve inputs from the vHPC to the anterior BNST (aBNST) nucleus, the number of aBNST-GAD67-positive cells is significantly reduced compared to that of a control group [44], suggesting a decrease in the activity of aBNST GABAergic neurons. Our study found that the numbers of GAD67-positive and c-Fos-positive cells in the amBNST and the level of *GAD67* mRNA were significantly reduced on D28 after the last MPTP injection, at a time when vDG excitatory synaptic transmission was already impaired. Moreover, the intra-vHPC D1R agonist could restore the activity of GAD67 expression and neuronal activation in the amBNST. These findings raise the possibility that vDG newborn neuron deficits, ameliorated by D1R activation, are associated with altered GAD67 expression and c-Fos immunoreactivity in the amBNST. It has been reported that reduced activity of hippocampal neurons and diminished BDNF secretion contribute to the suppressed firing of aBNST GABAergic neurons, accompanied by heightened stress sensitivity of the HPA-axis [39]. In MPTP-mice, the micro-injection of the AMPA into the amBNST or the muscimol into the PVN could have immediately mitigated the increase in HPA-axis activity.

**Figure 7:**
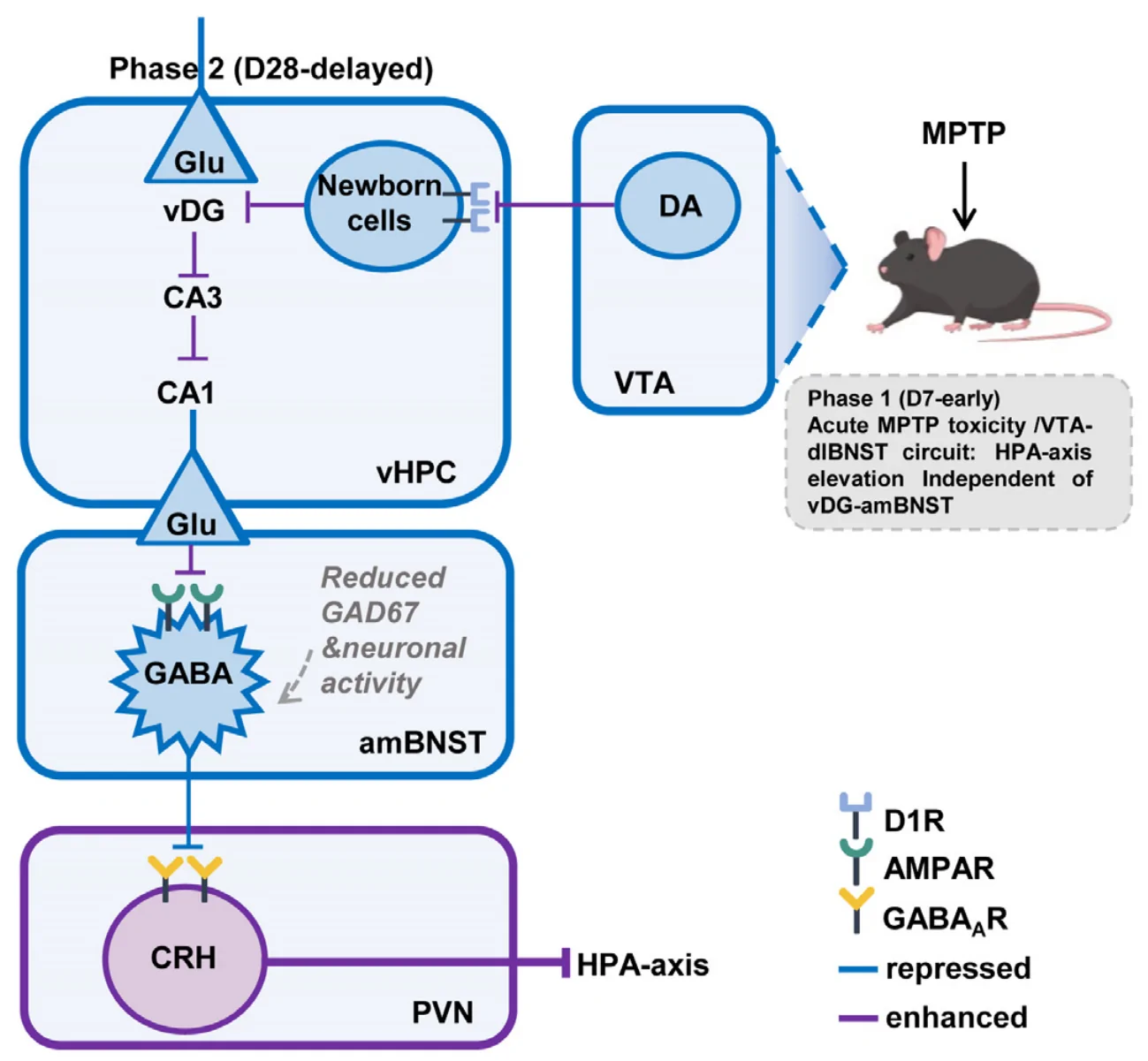
The hypothesis of molecular mechanisms underlying the MPTP-suppressed VTA DAergic neurons resulting in increased HPA-axis activity.

It should be noted that two-way ANOVA revealed no significant time-by-treatment interaction for CORT, ACTH, and PVN *CRH* mRNA, demonstrating that the magnitude of MPTP-triggered HPA-axis hyperactivity was statistically comparable between D7 and D28. Our two-phase conceptual model (Figure 7) is built upon pharmacological dissociation rather than temporal differences in the magnitude of HPA-axis elevation. This model distinguishes Phase 1 (D7, early) and Phase 2 (D28, delayed) based on our pharmacological observation: vHPC D1R agonist treatment rescues HPA-axis hyperactivity at D28 but not at D7. Since adult-born granule cells require approximately 4 weeks to mature and integrate into circuits, D1R-dependent rescue relying on newborn cell homeostasis would not be expected at the early D7 timepoint. At D7, newborn neurons are insufficiently mature to support circuit-level rescue, even though HPA-axis hormone levels are already elevated. The early D7 HPA elevation may stem from acute MPTP toxicity, peripheral sickness responses, repeated-injection stress, or direct VTA–dlBNST circuit disruption [15].

These results support a hypothetical cascade in which enhanced glutamatergic transmission in the vDG may modify molecular signals in amBNST GABAergic-phenotype neurons, which in turn may exert inhibitory control over the HPA-axis via GABA_A_R signaling.

### Limitations

Our study has several limitations. Firstly, repeated intra-vDG infusions may induce local tissue changes affecting neurogenesis measurements. Although cannulated vehicle controls matched for surgery and infusion were included, non-operated naive controls were absent. Our single BrdU-labeled approach cannot fully separate progenitor proliferation, neuronal differentiation and newborn cell survival; without BrdU/NeuN or BrdU/DCX co-staining, the neuronal identity of BrdU-positive cells cannot be definitively confirmed, and survival-related interpretations partly rely on prior published work [9]. Direct loss-of-function experiments selectively suppressing vDG neurogenesis were not performed, precluding strict causal proof linking vDG newborn cell deficits to amBNST-HPA-axis alterations. Secondly, the MPTP/MPP+ induces temporary, reversible VTA DAergic depletion, which does not recapitulate the progressive, irreversible loss of DAergic neurons characteristic of PD. Restoration of TH immunoreactivity and hippocampal DA does not equate to full functional recovery of DAergic circuits. We lack direct measurements of vHPC-amBNST glutamatergic and GABAergic synaptic transmission; circuit-level signaling inferences are based solely on indirect molecular and neuroendocrine markers. Of note, since GAD67 and c-Fos signals were assessed in separate tissue sections, reduced c-Fos activation cannot be specifically assigned to the GABAergic neuronal population, based on the data available. BDNF, a hypothesized downstream mediator, was not quantified. Furthermore, without anatomical tracing or vDG-restricted TH-fiber assessment, we cannot distinguish whether VTA influences on vDG are mediated by monosynaptic innervation or polysynaptic relays, and this interpretation depends on published anatomical data. Beyond pharmacological validation concerns, D1R-antagonist co-blocking experiments were not conducted to verify the receptor specificity of SKF38393-mediated effects; future co-administration of SKF38393 and SCH-23390 is needed to confirm D1R-dependent mechanisms. Another important consideration is that all experiments used only male mice. Given the known sex differences in HPA-axis regulation, hippocampal neurogenesis and BNST-related circuits, our results may not generalize to female animals.

## 5. Conclusions

Building on our previous findings that MPTP insult does not alter progenitor cell proliferation but compromises the survival of newly generated vDG neurons, we propose that disrupted D1R-dependent maintenance of newborn cell populations may compromise glutamatergic output from the vHPC, which could in turn alter amBNST function and drive concomitant changes in HPA-axis activity.

## Data Availability

The original contributions generated for this study are included in the article and Supplementary Materials. Further raw datasets are available from the corresponding author upon request.

## Supplementary Materials

The following supporting information can be downloaded at: https://www.mdpi.com/article/10.3390/brainsci16101098/s1, Figure S1: Effect of MPTP/MPP+ exposure on reference genes (GAPDH) for the RT-PCR. Bar graphs show levels of cycle thresh-old (Ct) values. (F_(3,20)_ = 0.1080, *p* = 180.9545, *n* = 6, one-way ANOVA).; Table S1: Summary of effect-size estimates and corresponding 95% confidence intervals for primary mechanistic statistical comparisons.

## Abbreviations

The following abbreviations are used in this manuscript:

aCSF	artificial cerebrospinal fluid
ACTH	adrenocorticotropic hormone
amBNST	anteromedial bed nucleus of the stria terminalis
ANOVA	analysis of variance
CORT	cortisol/corticosterone
CRH	corticotrophin-releasing hormone
CV	coefficient of variation
DAergic	dopaminergic
dHPC	dorsal hippocampus
DRs	DAergic receptors
fEPSP	excitatory postsynaptic potential
GABA	γ-aminobutyric acid
HPA	hypothalamic–pituitary–adrenal
HPLC-ED	high-pressure liquid chromatography coupled with electrochemical detection
IPIs	inter-pulse intervals
MPTP	1-methyl-4-phenyl-1,2,3,6-tetrahydropyridine
PD	Parkinson’s disease
PPF	paired-pulse facilitation
PVN	paraventricular nuclei
QC	quality control
SI	stimulating intensities
SPF	specific-pathogen-free
SNpc	substantia nigra pars compacta
TH	tyrosine hydroxylase
vDG	ventral hippocampal dentate gyrus
vHPC	ventral hippocampus
VTA	ventral tegmental area
