# Supplementary material for: MPTP-Induced D1R-Dependent Dysregulation of Ventral Hippocampal Newborn Cell Pools and Associated Alterations in the Hypothalamic–Pituitary–Adrenal Axis
Source: Brain Sci. 2026 Oct 12;16(10):1098. doi: 10.3390/brainsci16101098 (PMC13702120; doi:10.3390/brainsci16101098)
Supplement: Supplementary file 1 [file brainsci-16-01098-s001.zip › brainsci-4552929-supplementary.pdf]

Article

MPTP-Induced D1R-Dependent Dysregulation of Ventral Hippocampal Newborn Cell Pools and Associated Alterations in the Hypothalamic–Pituitary–Adrenal Axis

Tingting Di [1,2], Kai Zhang [1], Qian Ru [1], Wenxuan Yin [1], Yilin Jin [3], Ling Chen [4,*] and Hualing Li [1,2,*]

[1] School of Traditional Chinese Medicine, Faculty of Medicine, Yangzhou University, Yangzhou 225009, China; dididi@yzu.edu.cn (T.D.); 17715821816@163.com (K.Z.); mx320230562@stu.yzu.edu.cn (Q.R.); mx120231236@stu.yzu.edu.cn (W.Y.)
[2] Key Laboratory of the Jiangsu Higher Education Institutions for Integrated Traditional Chinese and Western Medicine in Senile Diseases Control, Yangzhou University, Yangzhou 225009, China
[3] The First School of Clinical Medicine, Faculty of Medicine, Yangzhou University, Yangzhou 225009, China; 232210616@stu.yzu.edu.cn
[4] Department of Physiology, Nanjing Medical University, Nanjing 211166, China
* Correspondence: lingchen@njmu.edu.cn (L.C.); hlli@yzu.edu.cn (H.L.)

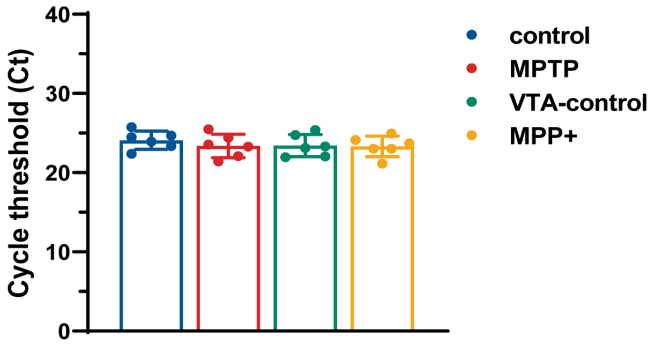

Figure S1. Effect of MPTP/MPP+ exposure on reference genes (GAPDH) for the RT-PCR. Bar graphs show levels of cycle threshold (Ct) values. ($F_{(3,20)}$ = 0.1080, p = 180.9545, n = 6, one-way ANOVA).

Table S1. Summary of effect-size estimates and corresponding 95% confidence intervals for primary mechanistic statistical comparisons.

Figure	F_value	df1	df2	Effect-size metric	CI_low	CI_high	Note
Figure 2b	15.68	2	15	0.67645	0.95	0.3835	
Figure 2c	8.635	2	15	0.53517	0.95	0.1838	
Figure 2f	23.95	2	15	0.76153	0.95	0.5289	
Figure 2g	10.73	2	15	0.58859	0.95	0.2526	
Figure 3d	15.19	3	20	0.69498	0.95	0.4544	7d
	9.745	3	20	0.59378	0.95	0.2986	28d
Figure 3e	12.34	3	20	0.64925	0.95	0.3816	7d
	8.988	3	20	0.57414	0.95	0.2708	28d
Figure 4c	0.443	5	36	0.05796	0.95	0	SI x treatment
	313.1	5	36	0.97752	0.95	0.9651	SI
	0.431	1	36	0.01183	0.95	0	treatment
Figure 4d	5.075	5	36	0.41344	0.95	0.148	SI x treatment
	99.79	5	36	0.9327	0.95	0.8952	SI
	61.1	1	36	0.62925	0.95	0.4606	treatment
Figure 4e	0.1165	2	18	0.01278	0.95	0	SI x treatment
	10.19	2	18	0.53101	0.95	0.2174	SI
	0.1891	1	18	0.0104	0.95	0	treatment
Figure 4f	8.051	3	12	0.66808	0.95	0.2813	
Figure 4g	0.0774	2	18	0.00852	0.95	0	SI x treatment
	15.19	2	18	0.62795	0.95	0.3471	SI
	0.8179	1	18	0.04346	0.95	0	treatment
Figure 4h	14.26	3	12	0.78094	0.95	0.5029	
Figure 5c	8.389	4	25	0.57306	0.95	0.2968	
Figure 5d	19.84	4	25	0.76044	0.95	0.589	
Figure 5e	7.37	4	25	0.54112	0.95	0.2524	
Figure 6c	6.738	2	15	0.47324	0.95	0.1149	7d
	9.955	4	25	0.61432	0.95	0.3568	28d
	0.00545	1	20	0.000272	0.95	0	time

	0.06335	1	20	0.003157	0.95	0	treatment
	17.99	1	20	0.473546	0.95	0.198	time x treatment
Figure 6d	8.171	2	15	0.52141	0.95	0.1675	7d
	18.63	4	25	0.74879	0.95	0.5696	28d
	0.00545	1	20	0.000272	0.95	0	time
	0.06335	1	20	0.003157	0.95	0	treatment
	17.99	1	20	0.473546	0.95	0.198	time x treatment
Figure 6e	4.807	2	15	0.39059	0.95	0.0416	7d
	6.743	4	25	0.51897	0.95	0.2228	28d
	0.05229	1	20	0.002608	0.95	0	time
	0.7695	1	20	0.03705	0.95	0	treatment
	18.89	1	20	0.485729	0.95	0.211	time x treatment
